# 

**Published:** 1960-01

**Authors:** 


### *Notes and News*

Professor R. Milnes Walker has been appointed a member of the Medical Research Council.

Dr. Percy Stocks has been awarded the Bisset Hawkins medal of the Royal College of Physicians.

Dr. Kenneth Bergin has been elected master of the Guild of Air Pilots and Air Navigators for 1959–60.

Obituary: Dr. J. E. Lucas.

### Appointments

### UNITED BRISTOL HOSPITALS

*May–November* 1959

| *Name* | *Appointment* | *Formerly* |
|---|---|---|
| LYON, D. G., PH.D., L.D.S., F.D.S.R.C.S. | Hon. Consultant Dental Surgeon (during tenure of post of Lecturer in Dental Surgery in the University of Bristol). | Senior Registrar status. |
| STEPHENSON, J. C., B.D.S., F.D.S.R.C.S., D.ORTH. | Senior Registrar in Orthodontics. | Senior Registrar, Eastman Dental Hospital. |
| SOLOMONS, D., M.B., B.CH., F.R.C.S.(EDIN.). | Registrar in Surgery. | Registrar, St. Catherine's Hospital, Birkenhead. |
| BHOWES, J. B., M.B., B.S. | Registrar in Anaesthetics. | S.H.O. in Anaesthetics, U.B.H. |
| WILKINS, J. L., M.B., B.S., D.C.H. | Registrar in Paediatrics. | Registrar in Paediatrics, South Devon and East Cornwall, H.M.C. |
| FRASER, I. D., M.B., CH.B. | Registrar in Pathology. | Resident Clinical Pathologist, B.R.I. |
| WILLS, M. R., M.B., CH.B. | Registrar in Pahtology. | Junior Clinical Pathologist, B.R.I. |

Dr. G. W. Burton, Lecturer in Anaesthetics in the University of Bristol, has been accorded the honorary status of Senior Registrar with the United Bristol Hospitals during the tenure of his University appointment.

Dr. K. R. Gough, Research Fellow in Medicine to the University of Bristol, has been accorded the honorary status of Registrar with the United Bristol Hospitals during the tenure of his University appointment.

### SOUTH WESTERN REGIONAL HOSPITAL BOARD

*August–November*, 1959

### BRISTOL

| *Name* | *Appointment* | *Formerly* |
|---|---|---|
| MISHRA, N., M.M.F. (CALCUTTA), D.T.M. & H. (ENG.). | Registrar in Diseases of the Chest, Bristol Chest Clinic/Ham Green. | J.H.M.O., Ham Green Hospital, Bristol. |
| APSIMON, H. T., M.B., CH.B. (BRISTOL), D.OBS.R.C.O.G. | Medical Registrar, Frenchay Hospital. | S.R.M.O., Frenchay Hospital. |

### BATH

| *Name* | *Appointment* | *Formerly* |
|---|---|---|
| CANNICOTT, S. M., M.A., M.B., B.CHIR.(CANTAB.) D.P.M. (CONJOINT) | Assistant Psychiatrist, Mendip Hospital, Wells. | Senior Registrar, Holloway Sanatorium. |
| NEATBY, G. M., M.A., M.R.C.S., L.R.C.P., D.O.M.S. | Ophthalmologist, Bath Clinical Area. | Clinical Assistant in Ophthalmology, Bath Eye Infirmary. |
| O'FLYNN, B.P., M.B., CH.B., B.A.O. (N.U.I.). | Registrar in Pathology to the Bath Group of Hospitals. | Registrar in Pathology, Bath Group of Hospitals. |

### SOUTH SOMERSET

| *Name* | *Appointment* | *Formerly* |
|---|---|---|
| AKEHURST, A. C., M.B., B.S.(LOND.), F.R.C.S. (ENG.). | Consultant General Surgeon, South Somerset Clinical Area. | Senior Surgical Registrar, B.R.I. |

### NORTH GLOUCESTERSHIRE

| | | |
|---|---|---|
| HART, R. J., M.A., M.B.B.S. (LOND.), F.R.C.S. (ENG.). | Consultant Orthopaedic Surgeon North Gloucestershire Clinical Area. | Senior Registrar, Royal National Orthopaedic Hospl. |
| LLOYD, T. W., D.M., B.CH.(OXFORD), M.R.C.P. (LOND.). | Consultant Physician Geriatrician, North Gloucestershire Clinical Area. | Physician Superintendent, St. Wulstan's Hospital. |
| WALKER, D. L., M.D.(LOND.). D.P.M. | Consultant Psychiatrist Medical Superintendent, Horton Road and Coney Hill Group of Hospitals. | Assistant Psychiatrist, (S.H.M.O.) Netherne Hospital, Coulsdon, Surrey. |
| BLATCHLEY, W. M.B.B.S.(LOND.) | Clinial Assistant to the Rheumatism Clinic in Gloucester. (Re-appointed.) | |

### DEVON AND CORNWALL

| | | |
|---|---|---|
| BRADBEER, T. L., M.B.B.S. (LOND.), F.R.C.S., D.L.O. (ENG.). | Consultant E.N.T. Surgeon, Devon and Exeter Clinical Area. | Senior Registrar, E.N.T. Department, Charing Cross Hospital. |
| WILSON, T. G., M.D.Ch.B., M.R.C.P. | Consultant Neurologist, Devon and Cornwall. | First Assistant to the Neurological Department, St. George's Hospital, S.W.1. |
| PARTRIDGE, J. P., M.B.B.S. F.R.C.S. | Consultant Surgeon, North Devon Infirmary, Barnstaple. | Consultant General Surgeon, Sydenham Children's Hospital, and Clinical Assistant, St. Mark's Hospital and the Hospital for Sick Children, London. |
| GOODEY, G. T., M.B., B.S. (LOND.), D.OBS.R.C.O.G. | Registrar in Obstetrics and Gynaecology, Royal Devon and Exeter Hospital. | Registrar in Obstetrics and Gynaecology, Whittington Hospital. |
| EADES, S. M., M.B., B.S. (LOND.). | Registrar in Paediatrics, South Devon and East Cornwall Hospital. | Working in Paediatric Department, Musgrove Park Hospital, Taunton. |
| JONES, J. O., M.B., Ch.B. (EDIN.). | Registrar in Psychiatry, Moorhaven Hospital, Plymouth. | S.H.O., Stratheden Hospital, Cupar, Fife. |
| ANDERSON, R. H., M.B.B.S. (LOND.). | Registrar in General Surgery, Royal Cornwall Infirmary, Truro. | S.H.O., General Surgery, Ashford Hospital, Middx. |
| WEEKS, MARY M., M.B., CH.B. | Assistant Geriatrician, West Cornwall. | Registrar in Geriatrics, Cowley Road Hospital, Oxford. |
| TEMPLETON, ANN, M.B., CH.B. (GLASGOW). | Assistant Medial Director, Mass Radiography Services, Devon and Cornwall. | — |
| STANLEY, H.W., M.B.B.S. (LOND.), D.A. | G.P. Anaesthetist, South Hams Hospital, Kingsbridge. | Locum General Practitioner, Salcombe, S. Devon. |
| THEODOROU, S., M.D. (ATHENS). | Registrar in Orthopaedic and Traumatic Surgery, Torquay and Exeter. | Registrar, Accident Unit, St. David's Hospital, Cardiff. |

